# Whether MTHFD2 plays a new role: from anticancer targets to anti-inflammatory disease

**DOI:** 10.3389/fphar.2023.1257107

**Published:** 2023-10-23

**Authors:** Hui Tang, Ning Hou

**Affiliations:** ^1^ Department of Pharmacy, Shandong Provincial Hospital Affiliated to Shandong First Medical University, Jinan, China; ^2^ Stem Cell Clinical Institute, Shandong Provincial Hospital Affiliated to Shandong First Medical University, Jinan, China

**Keywords:** methylenetetrahydrofolate dehydrogenase 2, T cells, inflammatory disease, therapeutic target, one-carbon metabolism enzyme

## 1 Introduction

Methylenetetrahydrofolate dehydrogenase 2 (MTHFD2) is a mitochondrial one-carbon (1C) metabolism enzyme that is overexpressed in cancer cells and barely expressed in most healthy adult tissues (Nilsson et al., 2014; Jha et al., 2023). The overexpression of MTHFD2 could provide the basis for biosynthesis of pyrimidine and purine during rapid proliferation of cancer cells which is widely needed for the growth of all tumors (Kim et al., 2016; Zhao et al., 2021; Bonagas et al., 2022; Zhao et al., 2022). Inhibition of MTHFD2 leads to imbalance of NADPH and redox homeostasis, which inhibits tumorigenic proliferation and growth, and increases cancer cell death under hypoxia (Ju et al., 2019). The knockdown of MTHFD2 leads to decreased expression of cell cycle genes suggesting interference with cell cycle progression (Yu et al., 2020). Because of the low expression of MTHFD2 in most adult tissues, targeting MTHFD2 is unlikely to produce significant side effects and MTHFD2 could be as a novel target for cancer therapy (Nishimura et al., 2019; Cuthbertson et al., 2021; Yang et al., 2021).

Recent research found that MTHFD2 was consistently overexpressed in many diseases, including ulcerative colitis, Celiac’s disease, systemic lupus erythematosus (SLE), psoriatic arthritis, Sjogren’s syndrome, multiple sclerosis (MS) and so on (Sugiura et al., 2022). Inhibition of MTHFD2 promotes regulatory CD4 T cell (Treg) activity, which suppresses the immune response. Does MTHFD2 play a new role from anticancer targets to inti-inflammatory disease?

## 2 A new role on anti-inflammatory disease and proposed mechanisms

In fact, what we are more interested in is that MTHFD2 deficiency can reduce disease degree in various inflammatory condition models. T-cell dependent Delayed Type Hypersensitivity (DTH) mouse models trials showed that MTHFD2 inhibitors did not increase inflammatory symptoms in mice, and increase animal weight, suggesting that the inhibitor has a protective effect on inflammation extending to B cell function (Sugiura et al., 2022). MS is an inflammatory demyelinating disease originating in the central nervous system. Compared to control group, Experimental Autoimmune Encephalomyelitis (EAE) model using with MTHFD2 inhibitors (MTHFD2i) resulted in significantly lower disease degree and cumulative clinical score. The infiltration of CD4st, CD4^+^ and CD8^+^ cells in the spinal cord of mice was significantly reduced after MTHFD2i treatment (Sugiura et al., 2022). In two other different inflammatory models-inflammatory bowel disease (IBD) and allergic airway disease, mice receptor of CD4^ΔMthfd2^ T cells continued gaining body weight, the number and frequency of CD4^ΔMthfd2^ T cells in spleen and mesenteric lymph nodes (MLNs) were significantly reduced. Meanwhile, the neutrophil richness in the bronchioalveolar lavage fluid (BALF) of CD4^ΔMthfd2^ mice affected by Alternaria-induced allergic airway disease showed a decreasing tendency (Sugiura et al., 2022). The sensitivity of T-cell to MTHFD2i might provide an efficacious strategy of immunotherapy for CD4^+^ T-cell-driven inflammation, and produce fewer adverse reactions than presently usable therapeutics. It should be worth studying whether T cell nucleus carries MTHFD2 and whether MTHFD2 is a therapeutic target for inflammatory disease.

CD4^+^ T cells are the key mediators and adaptive immunity which play a crucial role in host defense against pathogens (Candia and Matarese, 2022). CD4^+^ T cell subpopulations need MTHFD2 to varying degrees for activation, proliferation, survival, and cytokine production (Sugiura et al., 2022). Sugiura et al. (2022) have found that MTHFD2 in patients with inflammatory disease continues to upregulate combined with cell CRISPR-based screening and genetic test. The research showed that MTHFD2 may function as a metabolic checking point for the Th17/Treg cell axis and highlight its potential as a target for anti-inflammatory immunotherapeutic treatment. Meanwhile, MTHFD2i raised the basal and maximal oxygen consumption rate (OCR) of Th17 cells and decreased the expression of interferon-gamma (IFN-g) and interleukin (IL)-17 in Th1 and Th17 cells, which appears to alter the counterbalance between the pathogenic and anti-inflammatory state.

MTHFD2 has been shown to regulate *de novo* purine synthesis and signal transduction in activated T cells, promoting proliferation and the production of inflammatory cytokine (Ducker et al., 2016). MTHFD2 has been reported to transport to the nucleus and is presumed regulate gene expression (Gustafsson Sheppard et al., 2015). The lack of MTHFD2 could lead to the accumulation of intermediates in the purine synthesis pathway, which activates AMP-activated protein kinase to inhibit the mechanistic target of rapamycin (mTORC)1 (Su et al., 2019). The mTORC1 pathway plays a crucial role in promoting synthetic metabolism, driving a mass of the transcription factor ATF4 and inducing the expression of MTHFD2 (Ben-Sahra et al., 2016). Inhibition of mTORC1 signaling transduction might lead to changes in the metabolic process from glycolysis to mitochondrial respiration, and alter T cell receptor cycle metabolites (Shang et al., 2021).

## 3 Novel MTHFD2 inhibitors

**FIGURE 1:**
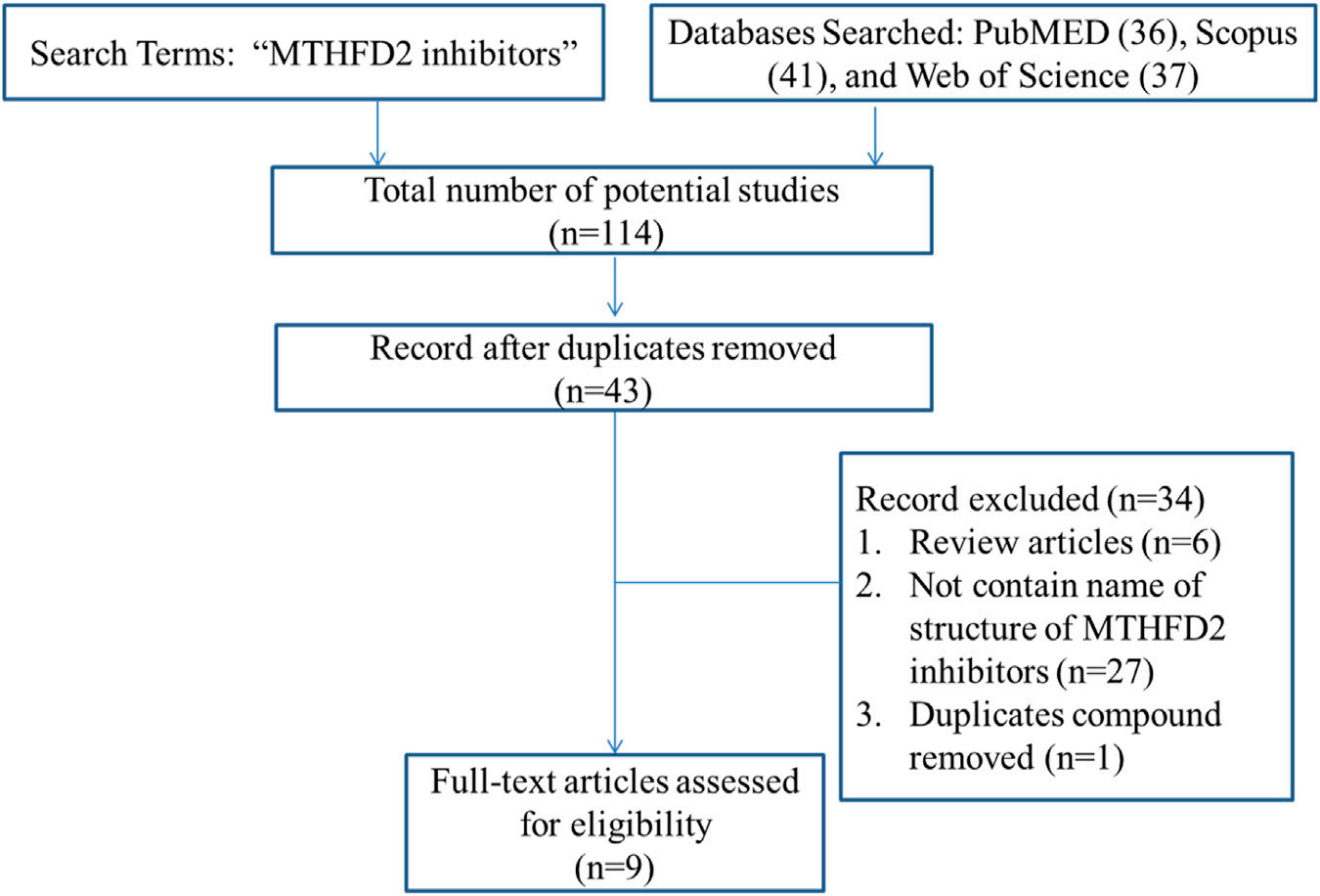
Study flow diagram of literature search.

One possible mechanism is that MTHFD2i damages T cell expansion through inadequate nucleotide production. Scientists have been working on the design and development of MTHFD2i as anticancer drugs (Table 1). Tricyclic coumarins and xanthine compounds are the only selective inhibitors of MTHFD2 reported to date (Jha et al., 2023). Comprehensive searches of English databases, including PubMed, Scopus, and Web of Science, and the time of index was from inception to 30 April 2023 for each database. Full-text searches were performed using “MTHFD2 inhibitors” in all fields (Figure 1). The dual MTHFD1/2 inhibitor LY345899 synthesized in 2017 has been demonstrated efficacy in improving disease conditions in the EAE model (Gustafsson et al., 2017; Ju et al., 2019). A simplification of the tricyclic core of LY345899 shows that TH9028, TH9619 and TH7299 are actually more active against MTHFD1 and MTHFD2L (Bonagas et al., 2022; Scaletti et al., 2022; Green et al., 2023). A novel isozyme-selective MTHFD2 inhibitor DS44960156 might provide further optimization options due to its >18-fold selectivity for MTHFD2 over MTHFD1, with a smaller molecular weight and favorable ligand efficiency (Kawai et al., 2019a). Subsequently, the same team developed an effective, selective, and oral MTHFD2i (DS18561882) which has favorable oral pharmacokinetic characteristics with the strongest cell activity and tumor growth inhibition (Kawai et al., 2019b; Lee et al., 2021). Most importantly, DS18561882 has been shown to reduce disease degree in variety of inflammatory disease models *in vivo* (Sugiura et al., 2022), which leads us to believe that MTHFD2 may be an anti-inflammatory and autoimmune target *in vivo* in the future.

**TABLE 1:** Novel MTHFD2 inhibitors.

Name	Target	Structure	Pathology
LY345899	Dual MTHFD1/2 inhibitor	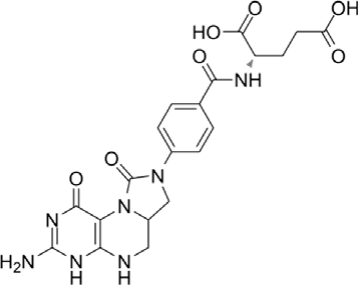	LY345899 treatment statistically significantly suppresses tumor growth and decreases the tumor weight in CRC patient-derived xenograft models
TH7299	Dual MTHFD1/2 inhibitor	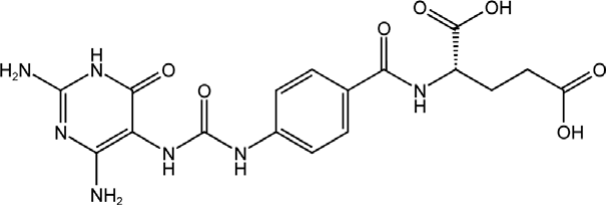	(−)
TH9028	Dual MTHFD1/2 inhibitor	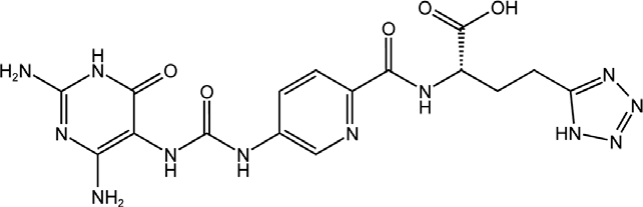	TH9028 and TH9619 showed overall strong antiproliferative efficacy in acute myeloid leukemia (AML) cells and T-ALL Jurkat cells comparable to standard-of-care compounds, with reduced effect on lymphoblastoid cell line (LCL) viability
TH9619	Dual MTHFD1/2 inhibitor	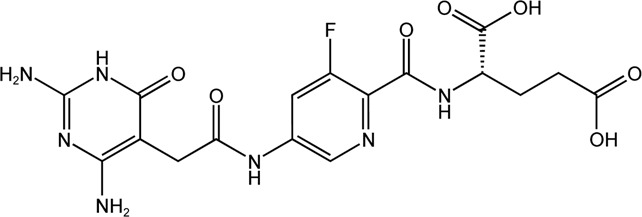	
DS44960156	MTHFD2 inhibitor	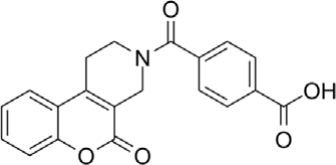	DS44960156has > 18-fold selectivity for MTHFD2 over MTHFD1, with a low molecular weight and a good ligand efficiency
DS18561882	MTHFD2 inhibitor	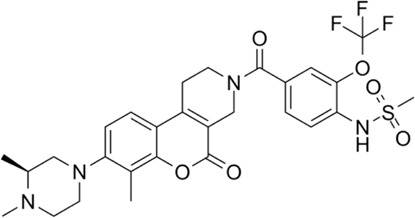	DS18561882, combined with enzalutamide can signifcantly inhibit CRPC cell proliferation *in vitro* and tumor growth *in vivo*. DS18561882 has also been shown to reduce disease degree in variety of inflammatory disease models *in vivo*

## 4 Conclusion

MTHFD2 is a mitochondrial one-carbon metabolism enzyme highly expressed in several human tumors, and targeting MTHFD2 has been used as the target of tumor therapy. Recent research suggests that MTHFD2 inhibitors appear to reduce inflammatory disease severity and alter the counterbalance between the pathogenic and anti-inflammatory state, which may serve as an anti-inflammatory and autoimmune target *in vivo* in the future. The research of anti-inflammatory drugs is expected to be promoted and developed.
